# Precision in treatment evaluation: importance of minimal clinically important differences (MCIDs) of outcome measures for autoimmune blistering diseases

**DOI:** 10.3389/fimmu.2023.1243581

**Published:** 2023-09-25

**Authors:** Henry Tseng, Corey Stone, Dédée F. Murrell

**Affiliations:** ^1^ Department of Dermatology, St. George Hospital, Sydney, NSW, Australia; ^2^ Faculty of Medicine, University of New South Wales, Sydney, NSW, Australia

**Keywords:** MCID, autoimmune blistering diseases, pemphigus, pemphigus disease area index, investigator global assessment, outcome measures

## Abstract

Autoimmune blistering diseases (AIBDs) comprise a group of rare conditions marked by autoantibodies that specifically target intercellular adhesion molecules. Despite the progress made in comprehending the disease and the increasing number of treatment options available, there is still no definitive cure for AIBDs such as pemphigus, and it continues to have a devastating impact on those affected. The challenges in achieving new approved therapies for AIBDs are complex and multifaceted. One significant obstacle was the prior lack of validated and standardized outcome measures, which are crucial for ensuring precise comparisons between new and traditional therapies. This gap in knowledge has prompted the development of minimal clinically important differences (MCIDs), which enable efficient and reliable comparison of therapeutic outcomes between trials. MCID is defined as the minimum difference in an outcome measure that indicates a clinically significant improvement/deterioration in disease severity. Additionally, MCIDs provide a patient-centered approach to evaluating treatment efficacy, by considering whether patients experience a subjective improvement in their symptoms. Therefore, this literature review will examine the derivation and significance of MCIDs for various scoring systems in AIBDs.

## 1 Introduction

This literature review will discuss the importance and derivation of MCIDs of various scoring systems in AIBD. The purpose is to understand the various methods which have been used to calculate MCIDs in AIBD and to recommend strategies to improve the reliability of MCID calculations.

## 2 Background

Autoimmune blistering diseases (AIBDs) encompass a group of rare conditions that result in the formation of blisters on the skin and mucous membranes, with examples including pemphigus and pemphigoid. The severity of AIBDs can be attributed to the fact that the rupturing of its blisters creates an opening for infections to opportunistically invade, putting the patient at increased risk of sepsis and ultimately death. This substantial infection risk is underscored by the high mortality rate of pemphigus, a type of AIBD, amounting to 1.7 to 3 times that of the general population (1). In addition to its poor outcomes, there is a shortage of international clinical trials exploring novel treatments for pemphigus that have the potential to significantly enhance the disease and the quality of life (QOL) of affected patients (2). This inadequacy can be attributed to the absence of validated and standardized outcome measures that are essential for making accurate comparisons between new and traditional therapies. To address this issue, minimal clinically important differences (MCIDs) of such outcome measures have been developed and used to allow for efficient comparison of therapeutic outcomes between trials. MCIDs not only facilitate comparison, but also provide a patient-centric approach to evaluating treatment efficacy, by taking into account whether the patient subjectively experiences an improvement in their symptoms (3).

## 3 Scoring systems for autoimmune blistering diseases

In medicine, the ability to accurately assess disease severity is often hindered by the lack of consensus on the best scoring systems to carry out such an assessment, which can lead to inaccurate assessments of patient treatment outcome and efficacy. This is especially true for AIBDs such as pemphigus before the development of its current scoring systems, as a systematic review conducted in 2006 highlighted the lack of universally recognized outcome measures as the contributing factor to the shortage of high-quality clinical trials assessing treatments for pemphigus (4). As a response to this issue, new scoring systems for the disease have since been developed and validated, including the two most-commonly used scoring systems in pemphigus: the Pemphigus Disease Activity Index (PDAI) and Autoimmune Bullous Skin Disorder Intensity Score (ABSIS) (5, 6).

Although assessment tools are valuable in dermatology, it is important to recognize that most of these clinician-reported outcome measures are designed to detect only small differences in severity. However, such minimal differences may not be significant enough to impact patients’ quality of life or measurable disease burden. Therefore, the concept of the MCID was created, which refers to the smallest difference in an outcome measure that reflects a clinically significant improvement in disease severity (7). Values above the MCID are used in clinical studies to show that a novel intervention is helpful for patients, which can be used in clinical practice to direct management.

### 3.1 How MCIDs are calculated

There are various different methods of calculating MCID which are summarized below in **Table 1**; however, by far the most common method is the anchor-based method because it directly takes into account the patients’ preferences and values (2). The anchor-based method compares score changes on a scoring system to an external “anchor,” which can be subjective patient-reported outcomes (e.g., QOL) or physician assessments, depending on the study context and outcome measure used (12).

**Table 1:** Methods used to calculate MCID: summary overview.

Method	Definition
**Anchor-based**	Using an external criterion (anchor) to identify a change in score that corresponds to a clinically significant improvement (2).
**Delphi method**	Uses expert opinion from a panel of experts who participate in a series of questionnaires to reach a consensus MCID. Responses from each round of questionnaires are fed back to the panel, and the experts are asked to revise their response. This process is repeated until a consensus opinion is reached (8).
Distribution-based methods	
Standard error of measurement (SEM)	Estimates the error associated with the measurement of a particular outcome. Criterion of achieving MCID: Patients who achieve an outcome score higher than the calculated SEM compared to those without or have stable disease (9).
Standard deviation (SD)	Using the number of standard deviations of the mean change score as criterion for achieving MCID. In practice, a change of 0.5 SD is most commonly used as a threshold for MCID (9). If the mean change score is at least 0.5 SD higher than the SD of the change scores, the treatment can be considered clinically significant.
Effect size (ES)	Measures the standardized difference between two groups, such as pre- to post-treatment, and calculating the magnitude of effect as standard deviation units (9, 10). The MCID is then calculated by determining the effect size that corresponds to a clinically meaningful improvement.
Minimal detectable change (MDC)	Smallest detectable change that is above the possible measurement error. The 95% confidence interval is commonly used for this (9).
Reliable change index (RCI)	Assesses whether the difference between two scores on a particular measure is statistically meaningful by dividing by the SEM. Threshold of above 1.96 is commonly used to determine clinical significance (11).
Standardised response mean (SRM)	Similar to effect size. Assesses magnitude of change following an intervention/treatment by dividing the mean change in scores with the standard deviation of the change in scores (10).

Once the anchor’s threshold of clinical significance is established, the MCID is then calculated as the smallest change in the scoring system that corresponds to a meaningful change in clinical improvement indicated by the anchor (12).

## 4 Previous MCIDs of scoring systems for autoimmune blistering diseases

The following section will explore the variations of methods that previous studies have employed to calculate MCIDs of AIBD.

### 4.1 PDAI

The PDAI is a scoring system developed by the International Pemphigus Definitions Committee over a period of three years after 2006, to evaluate the extent of pemphigus disease with a potential score ranging from 0-263 (5). It assesses activity and damage associated with the skin, scalp, and mucous membranes. The activity score is determined based on the numbers and sizes of erosions, blisters, or erythema observed during evaluation for each of the 12 anatomic locations. On the other hand, its damage score is determined by the presence (1 point) or absence (0 points) of post-inflammatory hyperpigmentation or erythema on resolving lesions on the skin and scalp (13).

To date, there have been no published studies on calculating the MCIDs for PDAI. The absence of this knowledge is likely because calculating MCID requires data on longitudinal changes in clinical scores in response to treatment from a reasonable sample size, which is difficult to collect and analyze for a disease as rare and severe as pemphigus (14). Determining the MCID for PDAI scores would offer valuable insights to clinicians and patients about the clinical significance of changes in PDAI scores over time. This knowledge can assist in guiding treatment decisions, providing realistic expectations for patients, and ultimately improving the care and quality of life of pemphigus patients.

### 4.2 ABSIS

The ABSIS is a scoring system that was initially developed for evaluating pemphigus but has since been widely used for all autoimmune blistering diseases following its introduction in 2007 (14, 15). The ABSIS has a possible score from 0-206 and assesses factors such as the patient’s body surface area affected by pemphigus, the location of the lesions on the skin and mucosa, as well as discomfort when eating or drinking designed to reflect the level of mucosal involvement (15). Furthermore, in contrast to the PDAI, the ABSIS incorporates the damage items into the overall score instead of treating them as individual components (16). Nevertheless, several multicenter studies have shown that the PDAI has higher interrater reliability than the ABSIS. For example, in a 2009 study conducted in the United States, the PDAI skin activity had an ICC of 0.86 compared to 0.39 for the ABSIS, demonstrating the superior reliability of the PDAI in comparison (17). In a 2012 study with 100 pemphigus vulgaris patients in Iran, the PDAI had the highest interrater reliability (ICC=0.98) among the scoring systems, followed by ABSIS (ICC=0.97) (18). The PDAI also correlated more strongly with disease extent and anti-desmoglein antibody levels, making it a more reliable tool for assessing pemphigus severity, especially in cases with variable cutaneous disease. In a recent multicenter international study with 116 pemphigus patients over 24 months (2019), both the PDAI and ABSIS showed strong interrater reliability at the beginning of the study. The PDAI had higher ICCs in moderate and extensive cases, while the ABSIS performed better in intermediate and extensive cases (16). Overall, such findings underscore that both the PDAI and ABSIS are valuable assessment tools for measuring pemphigus activity and suggest a preference for using the PDAI in multicenter studies.

There has been one study that calculated MCIDs for ABSIS led by Wijayanti et al. in 2017 (n=27) for bullous pemphigoid (14). The study used the anchor-based method by employing the receiver-operating characteristic (ROC) curve to calculate MCID values of BPDAI anchored against the Physician’s Subjective Assessment of Clinical Improvement (PSACI), a classification system that classifies disease activity as improved, stable, or deteriorated (2). Wijayanti et al. concluded that a 4.75-point decrease in BPDAI scores indicated clinical improvement (MCID=4.75), whereas a 4-point increase indicated clinical deterioration (MCID=4) (14). However, a significant limitation is its small sample size (n=27) which negatively affected the reliability and generalizability of its calculated MCID value due to the reduction in statistical power of analysis. In addition, the chosen anchor in the study assessed the severity of pemphigus from the physician’s rather than the patient’s perspective, which may not be reflective of how meaningful the change in severity is to the patient. Broadly classifying patients as clinically improved/stable/deteriorated is a relatively narrow categorization and may not capture the full range of disease activity/severity.

### 4.3 EBDASI

The Epidermolysis Bullosa Disease Activity and Scarring Index (EBDASI) is a scoring system that evaluates epidermolysis bullosa disease activity and damage separately in each of its five sections: skin, scalp, mucosa, nails, and other epithelialized surfaces. The skin section is the most comprehensive and includes 12 anatomical sites. The algorithm assigns a combined aggregate score of 506, with 276 for total activity and 230 for damage (19). The EBDASI has shown excellent reliability and validity as a scoring system for epidermolysis bullosa, as confirmed by its consistent performance in two separate Australian studies (19, 20).

There has been one study led by Jain et al. in 2016 (n=29) that likewise used the anchor-based method and ROC analyses to calculate MCIDs for EBDASI by anchoring against the 15-point Likert scale of change (21). A criterion of 3 on the Likert scale was chosen as the threshold for clinical significance, in accordance with various other MCID studies that have used the same established method. In their results, Jain et al. reported that a decrease of 7 or more points in the activity score indicated clinical improvement (MCID=7), while an increase of 3 or more points indicated clinical deterioration (MCID=3) (21). This study is similarly limited by its small sample size, although the anchor used provided broader categorization of disease activity across 15 points on the Likert scale of change.

### 4.4 BPDAI

The Bullous Pemphigoid Disease Area Index (BPDAI) is a scoring system developed in 2007 for bullous pemphigoid and is scored from 0-360 based on four components: body surface area percentage involved, peak pruritus numerical rating scale (NRS) score, disease extent index, and area index (22). The pruritus component is considered as a subjective aspect of the BPDAI and is evaluated separately from the scores of the other three components (23). In addition, a recent multicenter study in Europe (2021) reaffirmed the reliability of the BPDAI as a robust tool for assessing bullous pemphigoid (BP) severity, which demonstrated a high baseline intraclass correlation coefficient (ICC=0.97) that remained stable up to month 6 (22). The study involved 285 bullous pemphigoid patients from 50 dermatology departments and established cut-off values for categorizing mild, moderate, and severe bullous pemphigoid cases based on BPDAI score percentiles. The calculated cut-off values were 20, 57, and above 57, respectively. Furthermore, the improvement in BPDAI scores correlated with a decrease in anti-BP180 antibodies but not with anti-BP230 antibodies (22). These findings highlight the BPDAI’s precision in assessing BP severity and provide valuable clinical classification cut-off values.

In Wijayanti et al.’s same study in 2017 which also calculated MCIDs for ABSIS, Wijayanti et al. concluded that a 4-point decrease in BPDAI scores indicated clinical improvement (MCID=4), whereas a 3-point decrease indicated clinical deterioration (MCID=3) (14). In addition, Wijayanti et al. also concluded that the BPDAI was a reliable scoring system in assessing bullous pemphigoid severity, demonstrating both strong interrater and intrarater reliability (14).

### 4.5 IGA

The investigator global assessment (IGA) for pemphigus is a new scoring system that is still in the process of being developed and validated by a previous study as of this writing.

The IGA is scored separately from 0-4 (0=clear; 4=severe) for mucosal and cutaneous lesions, making it more simplistic than PDAI and ABSIS scores. The U.S. Food and Drug Administration (FDA) prefers to use the IGA to assess the primary endpoint of clinical drug trials over the PDAI and ABSIS because the IGA provides a more simplistic and clinically meaningful ordinal score that patients can easily understand (24). Developing and calculating the MCID of a validated IGA for pemphigus will thus aid clinical trials and development of new treatments for pemphigus.

### 4.6 MCIDs of other commonly used scoring systems in dermatology

The variation in methods which have been used to calculate MCIDs highlighted above underscores the inconsistency of calculating MCIDs of scoring systems for AIBD. This appears to be a common theme for many other scoring systems in dermatology (3). The following table (**Supplementary Table 1**) compares the various methods used to calculate MCID of commonly used dermatological scoring systems and their advantages and disadvantages.

### 4.7 The credibility of MCID calculations: an analysis

The inconsistency in calculating MCIDs is evident from the various methods used, as highlighted in the discussion so far. The absence of international guidelines for calculating MCIDs leads to varying sample sizes in studies, which are often on the low end for rarer and more severe diseases like pemphigus. This has negative consequences on the generalizability of MCIDs. It is known that the MCID may exhibit variability based on differences in population and context, and to address this issue, it is imperative to accept that MCIDs can and should be re-calculated should the opportunity arise for sample sizes to be expanded (25, 26).

In addition, from the examples above, it is evident that MCIDs vary with the choice of anchor. Given that MCIDs reflect the level of improvement from the *patient’s* perspective, it appears to be logical that anchoring to a score that patients give themselves, such as QOL scores, would be more favorable than a physician’s score such as the 15-point Likert scale of change. However, in as much as 41.4% of MCID calculations in dermatology, the anchor was rated by the physician, though there are advantages and disadvantages to both (3). One advantage to having an anchor scored by a physician is ensuring that the MCID calculated reflects an objective improvement in patient’s condition particularly when assessing disease activity, but a disadvantage is the inability of the MCID to reflect a change felt by the patient. It can be argued that having a physician-reported anchor is better suited for severe and potentially life-threatening conditions such as pemphigus as any small change in disease activity would be significant to the patient’s ongoing management more so than whether the patient feels he/she has improved. In addition, patient perception of their level of improvement in disease severity may be skewed by other, unrelated factors such as treatment side effects (27). These factors could potentially bias results of the calculation of MCIDs should patient-reported anchors be used.

### 4.8 Implications for future MCID calculations

Overall, the inconsistency of MCID calculations identified arises from the use of various methods and anchors in different studies. The absence of standardized guidelines leads to varying sample sizes, particularly in rare and severe diseases, which affects the generalizability of MCIDs.

To address this issue, it is crucial to recognize that MCIDs can and should be recalculated when opportunities arise to expand sample sizes. To improve the credibility of MCID calculations in dermatology, it is recommended to establish international guidelines that provide standardized methods for calculating MCIDs. These guidelines should address issues such as sample size determination and the selection of appropriate anchors based on the specific disease, its severity, and objectives of the studies. The selection of appropriate anchors for MCID calculations should not only consider the specific disease and its severity but also take into account the objectives of the studies. Different objectives may require different types of anchors to be used in calculating MCIDs. For instance, studies aiming to assess the impact of treatments on disease severity may use patient-reported anchors, whereas studies focusing on clinical significance may opt for physician-reported anchors. By acknowledging the influence of study objectives on anchor selection, researchers can ensure that MCID calculations align more closely with the intended applications of the study results. Furthermore, collaboration among researchers and clinicians can facilitate the pooling of data and resources to achieve larger sample sizes, enhancing the accuracy and generalizability of MCIDs. Overall, by promoting consistency and transparency in the calculation of MCIDs, the reliability and usefulness of these values in dermatology research and clinical practice can be enhanced.

## 5 Conclusion

The current inconsistencies in MCID calculations underscore a significant gap in literature in this area. The determination and application of MCIDs in AIBD are essential for evaluating treatment effectiveness and enhancing patient care. By calculating MCIDs through anchor-based and distribution-based approaches, clinicians can gain insights into clinically significant improvements and make informed treatment decisions. Further research is needed to establish MCIDs for different scoring systems using both patient- and physician-reported anchors and enhancing sample sizes to improve the application of MCID calculations in dermatology.

## Author contributions

HT wrote the original manuscript. CS and DFM have made a substantial contribution revising the manuscript critically related to the accuracy or integrity of any part of the work. All authors contributed to the article and approved the submitted version.
