# Supplementary material for: Precision in treatment evaluation: importance of minimal clinically important differences (MCIDs) of outcome measures for autoimmune blistering diseases
Source: Front Immunol. 2023 Sep 25;14:1243581. doi: 10.3389/fimmu.2023.1243581 (PMC10560849; doi:10.3389/fimmu.2023.1243581)
Supplement: Supplementary file 1 [file Table_1.docx]

Supplementary Material

Precision in Treatment Evaluation: Importance of Minimal Clinically Important Differences (MCIDs) of Outcome Measures for Autoimmune Blistering Diseases

Henry Tseng^1,2*^, Corey Stone^1,2^, Dédée F Murrell^1,2*^

^1^ Department of Dermatology, St. George Hospital, Sydney, NSW, Australia

^2^ Faculty of Medicine, University of New South Wales, Sydney, NSW, Australia

**Orchid IDs:**

- **Henry Tseng: 0009-0004-8074-3759**
- **Corey Stone: 0000-0002-4847-8447**
- **Dédée F Murrell: 0000-0003-2971-0199**

*** Correspondence:**Initial corresponding author: Henry Tseng
z5277936@unsw.edu.au

Final corresponding author (post-publication): Professor Dédée Murrell
d.murrell@unsw.edu.au

| **Scoring System** | **Author** | **Dermatological Condition** | **Method Used** | **Anchor** | **Findings** | **No. of patients** | **Advantages** | **Disadvantages** |
| --- | --- | --- | --- | --- | --- | --- | --- | --- |
| EBDASI^38^ | Jain, S. V., Harris, A. G., Su, J. C., Orchard, D., Warren, L. J., McManus, H., & Murrell, D. F. (2017) | Epidermolysis bullosa | Anchor-based method of ROC analyses | 15-point Likert scale of change | Calculated MCID was 9-point reduction for clinically significant improvement and 3-point increase for deterioration. EBDASI demonstrated high inter- and intra-observer reliability and was responsive to changes in disease activity. | 29 | Pilot study to calculate MCID for EBDASI. MCIDs calculated were determined to have high sensitivity, specificity, and percentage correct classification. Physician anchor was consistently scored by the same physician, which increases confidence. | Small number of patients for MCID calculation due to the rarity of EB. Utilised objective, physician-scored anchor (Likert scale), which may not be reflective of patient perspective of clinical improvement. Given heterogenicity of EB, MCIDs may not be reflective of all patients. |
| BPDAI and ABSIS^35^ | Wijayanti, A., Zhao, C. Y., Boettiger, D., Chiang, Y. Z., Ishii, N., Hashimoto, T., & Murrell, D. F. (2017) | Bullous Pemphigoid | Anchor-based method of ROC analyses | Physician’s Subjective Assessment of Clinical Improvement (PSACI), a classification system of disease activity as improved, stable, or deteriorated | Calculated MCID for ABSIS was 8.6-point reduction for clinically significant improvement and 4-point increase for deterioration. For BPDAI, MCID was 4 for improvement and 3 for deterioration. Both BPDAI and ABSIS are reliable and valid disease scores with good responsiveness to clinical change in BP. | 27 | Pilot study to calculate MCID for BPDAI. Similar to Jain et al.'s study, this study also evaluated the sensitivity, specificity, and percentage correct classification of MCID scores. | Small number of patients for MCID calculation for a similar reason given rarity and severity of BP. Anchor used (clinically improved/stable/deteriorated) has relatively narrow categorisation and may not capture the full range of disease activity/severity. |
| EASI, SCORAD (SCORing Atopic Dermatitis), POEM (Patient Orientated Eczema Measure)^42^ | Schram, M. E., Spuls, P. I., Leeflang, M. M. G., Lindeboom, R., Bos, J. D., & Schmitt, J. (2012) | Atopic Dermatitis | Anchor-based method of ROC analyses | vIGA atopic dermatitis | Calculated MCID for EASI was ± 6.6 for clinical improvement/deterioration. For SCORAD, MCID was ± 8.7. For POEM, MCID was ± 3.4. | 42 | First of two studies to calculate MCID for EASI scores to date. Moderate number of patients. | Correlation between calculated MCID scores and percentage correct classification is missing. Sample size could have been larger given frequency of atopic dermatitis in the community. Anchor used (IGA) is likewise physician reported. |
| vIGA-AD^43^ | Simpson, E. L., Bissonnette, R., Paller, A. S., King, B., Silverberg, J. I., Reich, K., Thyssen, J. P., Doll, H., Sun, L., DeLozier, A. M., Nunes, F. P., & Eichenfield, L. F. (2022) | Atopic Dermatitis | Anchor-based method of ROC analyses | Patient Global Impression of Severity – Atopic Dermatitis (PGI‐S‐AD) | vIGA‐AD change of -1.0 as clinically meaningful. | 853 | Pilot study to calculate MCIDs for the vIGA-AD. Large sample size. Utilised patient-reported anchor. Multinational study. | Correlation between calculated MCID scores and percentage correct classification is missing. |
| CDASI-A (Cutaneous Dermato- myositis Area and Severity Index Activity)^44^ | S. Ahmed, S. Chakka, J. Concha, R. Krain, R. Feng, V.P. Werth (2020) | Dermatomyositis | Anchor-based method and linear regression models | Skindex-29 (QOL measure in 3 categories: Functioning, Emotions, and Symptoms) | Calculated MCID for CDASI-A was 7.86 for Symptoms and 10.29 for Emotions for clinical improvement/deterioration. | 103 | Anchor used strongly reflects QOL (patient reported). Moderate number of patients. | Correlation between calculated MCID scores and percentage correct classification is missing. Linear regression model assumes linear relationship between anchor and CDASI-A, which may not always be the case. |
| EASI, SCORAD, POEM^45^ | Silverberg, J. I., Lei, D., Yousaf, M., Janmohamed, S. R., Vakharia, P. P., Chopra, R., Chavda, R., Gabriel, S., Patel, K. R., Singam, V., Kantor, R., & Hsu, D. Y. (2021) | Atopic Dermatitis | Anchor-based method of ROC analyses | Patient‐Reported Global Assessment of AD severity (PtGA) | Calculated absolute MCID for EASI was 2.7 to 15.8 for mild AD, 17.5 to 23.3 for moderate AD, and 22.3 to 29.2 for severe AD. | 826 | Most recent MCID study on EASI scores. High number of patients. Anchor used was patient reported (PtGA). Reported different MCIDs for different AD severities, highlighting that MCID evolves over time. | Correlation between calculated MCID scores and percentage correct classification is missing. |
| CLASI-A (Cutaneous Lupus Disease Area and Severity Index Activity Score)^46^ | Chakka, S., Krain, R. L., Ahmed, S., Concha, J. S. S., Feng, R., Merrill, J. T., & Werth, V. P. (2022) | Cutaneous lupus erythematosus | Anchor-based method and linear regression models | Skindex-29 | Calculated MCID for CLASI-A was ≥5 for Symptoms and ≥7 for Emotions for clinical improvement/deterioration | 126 | Anchor used strongly reflects QOL (patient reported). Moderate number of patients. | Correlation between calculated MCID scores and percentage correct classification is missing. Linear regression model assumes linear relationship between anchor and CLASI-A, which may not always be the case. |
| Review of scoring systems^2^ | Hanna, S., Kim, M., & Murrell, D. F. (2016) | Pemphigus | N/A | N/A | Provides an overview of the different scoring systems for pemphigus, including MCIDs and validation measures. | N/A | A comprehensive review and evaluation of various outcome measures used to assess severity and activity in pemphigus as well as comparison to other outcome measures in dermatology. |  |

**Supplementary Table 1.** Review of Literature on MCIDs of Commonly Used Scoring Systems in Dermatology
